# Plasma R-spondin 2 levels are associated with the progression of diabetic kidney disease

**DOI:** 10.3389/fendo.2026.1787296

**Published:** 2026-03-12

**Authors:** Yuren Wang, Weiyuan Chen, Xin Xiong, Jiaran Zhu, Qingshan He, Linlin Zhang, Hua Qu, Hongting Zheng, Yi Zheng

**Affiliations:** 1 Department of Endocrinology, Metabolic and Chronic Disease Science Innovation Center, The Second Affiliated Hospital of Army Medical University, Chongqing, China; 2 The Second Affiliated Hospital of Guizhou University of TCM, Guiyang, China

**Keywords:** biomarker, diabetic kidney disease, kidney injury, RSPO2, T2DM

## Abstract

**Aims:**

Circulating factor R-spondin 2 (RSPO2) has been found to play a role in lipid metabolism and insulin resistance. However, its relationship with kidney injury and diabetic kidney disease (DKD) remains unclear. We aim to determine the expression levels of RSPO2 in patients with type 2 diabetes mellitus (T2DM) stratified by kidney disease risk, and analyze its correlations with the parameters of renal injury parameters, glucose and lipid profiles, and further investigate the association between plasma RSPO2 concentration and the prognosis risk of DKD.

**Methods:**

A total of 121 participants with T2DM were enrolled and categorized into four kidney disease risk groups (Low, Moderately Increased, High, and Very High Risk) based on estimated glomerular filtration rate (eGFR) and urine albumin-to-creatinine ratio (UACR). Relevant anthropometric and biochemical parameters were analyzed, and plasma RSPO2 concentrations were measured. Spearman’s correlation analysis and Multiple logistic regression analysis was conducted.

**Results:**

Plasma RSPO2 concentrations were significantly elevated in the High and Very High Risk groups compared to the Low and Moderately Increased Risk groups. *In vitro*, RSPO2 expression levels were elevated in kidney tissues of DKD mouse models. Spearman correlation analysis revealed that the plasma RSPO2 level was positively correlated with UACR, Urea, serum creatinine and history of hypertension, while negatively correlated with eGFR, diastolic blood pressure (DBP), drinking and metformin usage after adjusting for age. Multivariate logistic regression analysis demonstrated that a higher plasma RSPO2 concentration remained significantly associated with progression to a more severe kidney disease risk grade after adjusting for potential confounders.

**Conclusions:**

Elevated plasma RSPO2 concentration is independently associated with higher kidney disease risk in patients with T2DM and may serve as a potential biomarker for DKD progression. Further studies are needed to elucidate the role of RSPO2 in DKD and its underlying mechanisms.

## 1 Introduction

Currently, there are roughly 529 million people with diabetes worldwide (1). Among which approximately 40% of type 2 diabetes mellitus (T2DM) and 30% of type 1 diabetes mellitus (T1DM) patients may develop diabetic kidney disease (DKD) (2). DKD has become the main cause of chronic kidney disease (CKD) and end-stage renal disease (ESRD). From epidemiological data, it is estimated that the 10-year all-cause mortality rate for diabetic patients with CKD is 31.1% (2). Hence, it’s crucial to identify key risk factors linked to DKD progression for early disease prediction.

Recent studies have highlighted circulating factors as key risk factors for diabetic complications. For example, significantly elevated plasma C-C motif ligand 7 (CCL7) levels have been reported in T2DM patients, and inhibition of CCL7 markedly alleviates diabetes-induced vascular endothelial dysfunction and vasculopathy (3). Moreover, serum levels of fibroblast growth factor 1 (FGF1) were decreased and positively correlated with fraction shortening in diabetic cardiomyopathy patients, and treatment with a FGF1 variant with reduced proliferative potency prevented diabetes induced cardiac injury and remodeling and restored cardiac function (4). Thus, it is essential to identify key risk factors associated with disease progression to predict DKD advancement, with future studies aimed at validating and intervening in these factors to explore their potential as pathogenic agents and therapeutic targets.

The family of R-spondin secreted ligands (RSPO) 1–4 act via their cognate leucine rich repeat containing G protein-coupled receptor (LGR) to amplify the canonical Wnt signaling, having a significant regulatory effect on embryo development, tissue homeostasis, as well as disease occurrence (5–7). Among the four members, RSPO1, RSPO2, and RSPO3 have been reported to be associated with glycolipid metabolism, while RSPO4 is the main pathogenic gene for inherited anonychia (8–11). Specifically, serum RSPO1 has been confirmed as a new surrogate marker for obesity and insulin resistance (12). More recently, Dong et al. found that increased circulating RSPO2 levels correlate with impaired glucose homeostasis in male obese individuals, and lead to adipose tissue hypertrophy and insulin resistance in mice (13). Blocking the binding of RSPO2 to LGR4 increases energy expenditure by promoting the browning of white fat, conferring resistance to both diet-induced and genetic obesity (14). Additionally, RSPO2 inhibits hepatic steatosis (15) and participates in the pathogenesis of atherosclerosis (16). Similarly, elevated plasma levels of RSPO3 are associated with an increased risk of T2DM. Notably, RSPO1 knockdown has been found to alleviate obesity-related kidney dysfunction and renal fibrosis (17). RSPO3 recently has been identified as associated with CKD in individuals with abnormal glucose metabolism (18). However, RSPO2 function in DKD remains to be elucidated.

We conducted a cross-sectional study to evaluate plasma RSPO2 levels in patients with T2DM across varying levels of kidney disease risk, analyze its correlation with renal function and injury parameters, and further explore its impact on the prognosis risk of DKD.

## 2 Materials and methods

### 2.1 Study subjects

A total of 121 patients with T2DM were recruited based on the 1999 World Health Organization (WHO) diagnostic criteria (19), which requires a fasting plasma glucose ≥ 7.0 mmol/L, a 2-hour plasma glucose during a 75-g oral glucose tolerance test ≥ 11.1 mmol/L, or a random plasma glucose ≥ 11.1 mmol/L in the presence of classical hyperglycemic symptoms, with all criteria requiring confirmation on at least two separate occasions. The classification as T2DM was subsequently made based on adult-onset presentation and the absence of indicators of T1DM. DKD was diagnosed in case of estimated glomerular filtration rate (eGFR) lower than 60 mL/min/1.73 m^2^ and/or elevated urinary albumin creatinine ratio (UACR) ≥ 30 mg/g albumin creatinine observed for a minimum of 3 months. According to the Kidney Disease: Improving Global Outcomes (KDIGO) risk prognosis grid (20), which stratifies prognostic risk for future kidney disease outcomes, individuals were categorized into four prognostic risk groups based on their individual eGFR and UACR values: (1) Low Risk: eGFR ≥ 60 mL/min/1.73 m^2^ and UACR < 30 mg/g. (2) Moderately Increased Risk: eGFR ≥ 60 mL/min/1.73 m^2^ and UACR 30–300 mg/g, or eGFR 45–59 mL/min/1.73 m^2^ and UACR < 30 mg/g. (3) High Risk: eGFR 30–44 mL/min/1.73 m^2^ and UACR < 30 mg/g, or eGFR 45–59 mL/min/1.73 m^2^ and UACR 30–300 mg/g, or eGFR ≥ 60 mL/min/1.73 m^2^ and UACR > 300 mg/g. (4) Very High Risk: eGFR < 30 mL/min/1.73 m^2^, or eGFR 30–44 mL/min/1.73 m^2^ and UACR 30–300 mg/g, or UACR > 300 mg/g with eGFR < 60 mL/min/1.73 m^2^. Inclusion criteria were as follows: (a) age > 18 years; (b) diagnosis of T2DM as defined above. Exclusion criteria were as follows: (a) acute diabetic complications, including diabetic ketoacidosis, hyperosmolar hyperglycemic status, or diabetic lactic acidosis; (b) CKD attributable to other causes; (c) significant comorbidities such as liver disease, coronary heart disease, or cerebrovascular disease; (d) current systemic corticosteroid treatment; (e) pregnancy or lactation. The studies involving human participants were reviewed and approved by the Ethics Committee of Xinqiao Hospital, Army Medical University. Written informed consent to participate in this study was provided by the participants or their next of kin. This study was prospectively registered online (Clinical Trial Registration No. ChiCTR-ROC-17010719) and conducted in compliance with the principles outlined in the Declaration of Helsinki.

### 2.2 Clinical data and sample collection

Demographic characteristics, clinical parameters, and medical history were obtained from the electronic medical record system at Xinqiao Hospital. Collected data included age, sex, height, weight, smoking and alcohol consumption history, history of hypertension, duration of diabetes, serum creatinine (Scr), urinary creatinine (Ucr), urinary microalbumin (u-mAlb), eGFR, uric acid (UA), Urea, systolic blood pressure (SBP), diastolic blood pressure (DBP), fasting blood glucose (FBG), glycated hemoglobin (HbA1c), low-density lipoprotein cholesterol (LDL-C), high-density lipoprotein cholesterol (HDL-C), total cholesterol (TC), triglycerides (TG), and history of diabetes-related medication use. Body mass index (BMI) was calculated as weight (kg)/height (m²). UACR (mg/g) was calculated as u-mAlb (mg/L)/Ucr (g/L). Diabetes duration was defined as the time from diagnosis to study inclusion. Hypertension history was defined as a prior clinical diagnosis or use of antihypertensive medications. Diabetes-related medication included metformin, sulfonylureas, alpha-glucosidase inhibitors, insulin, dipeptidyl peptidase-4 (DPP-4) inhibitors, sodium-glucose cotransporter-2 (SGLT2) inhibitors, and glucagon-like peptide-1 receptor (GLP-1R) agonists. HbA1c was measured using the BIO-RAD VARIANTTM II & D-10TM system (Hercules, CA, USA). FBG, TC, TG, LDL-C, HDL-C, UA, Urea, Scr, Ucr and u-mAlb were measured using the Beckman CX-7 chemistry analyzer (Brea, CA, USA). After an overnight fast, peripheral venous blood samples were collected the next morning, centrifuged at 3000×g for 15 min at 4 °C, aliquoted, and stored at -80 °C. Morning urine was collected, aliquoted, and similarly. Cryopreserved samples were used within 3 months.

### 2.3 Plasma RSPO2 concentration determination

Plasma RSPO2 concentration was quantified using a commercially enzyme-linked immunosorbent assay (ELISA) kit (CSB-EL020551H, Wuhan Huamei Biotech Co., Ltd., Hubei, China), with a detection range of 0.156–10 ng/mL and a sensitivity of 0.039 ng/mL. All samples were diluted twofold. Intra- and inter-assay coefficients of variation were <8% and < 10%, respectively. Absorbance was measured at 450 nm using a spectrophotometer, with all assays performed in duplicate. Concentrations were derived from the standard curve.

### 2.4 Mice

Male C57BL/6, BKS-db/db and BKS-db/m mice were purchased from Shanghai Model Organisms Center, Inc. T1DM was induced in 8-week-old C57BL/6J mice by intraperitoneal injection of streptozotocin (STZ, Sigma-Aldrich) (50 mg/g, pH 4.5, dissolved in sodium citrate) for 5 consecutive days. Successful modeling was confirmed by a fasting blood glucose > 13.8 mM two weeks post-injection. For the T2DM model, 8-week-old BKS-db/db mice were selected, with BKS-db/m as controls. All mice were housed under SPF conditions at 25 ± 1 °C, 55 ± 10% humidity, with a 12-hour light/dark cycle and free access to food and water. At the end of the study, mice were injected with sodium pentobarbital (100 mg/kg i.p.) for anesthesia, followed by cervical dislocation for euthanasia, and kidney tissues were collected for ex vivo analysis. All animal experiments were conducted in accordance with the protocols approved by the Laboratory Animal Welfare and Ethics Committee of the Army Medical University (AMUWEC20235154). This study adhered to the Laboratory Animal Welfare Guidelines (GB/T 35892-2018, International Standard Classification Number: 65.020.30).

### 2.5 Quantitative real-time PCR

Quantitative real-time PCR was performed as previously described (21). Primer sequences: RSPO2, 5′-TGCCTCTTCTCATTTGCCCT-3′ (F) and 5′-GGGATTTGATACATAACTAGCT CGC-3′ (R); GAPDH, 5′-TGAACGGGAAGCTCACTG-3′ (F) and 5′-TCCACCACCCTGTT GCTG-3′ (R).

### 2.6 Western blots

For protein extraction, tissue lysates were prepared as previously described (22). Protein concentrations were measured using the BCA Protein Assay Kit (Beyotime). Extracted protein lysates were resolved by SDS-PAGE and immunoblotted with the indicated primary antibodies (1:1000) and their corresponding HRP-conjugated secondary antibodies. Blots were developed with chemiluminescent HRP substrate (Millipore) and imaged using a fusion FX5s system (Vilber Lourmat). The anti-RSPO2 was obtained from Bioss (bs-18876R). AntiGAPDH antibody was obtained from Aksomics (Shanghai).

### 2.7 Statistical analyses

Statistical analyses were conducted using SPSS 24.0 (IBM Corp, NY, USA), and data visualization was conducted with GraphPad Prism (version 9.5). The normality of continuous variables was evaluated using the Kolmogorov-Smirnov test. Normally distributed data are presented as mean ± standard deviation (SD), and compared between groups using one-way analysis of variance (ANOVA) when the assumption of homogeneity of variances was met (Levene’s test *P* ≥ 0.05), followed by appropriate *post hoc* tests if the ANOVA reached statistical significance (*P* < 0.05). If the assumption of homogeneity of variances was violated (Levene’s test *P* < 0.05), Tamhane’s T2 method was applied for multiple comparisons. Non-normally distributed variables are presented as median (interquartile range, IQR) and compared using the Kruskal-Wallis H test. If a significant difference was detected (*P* < 0.05), *post hoc* Mann-Whitney U tests with Bonferroni correction were performed for pairwise comparisons. Categorical variables are presented as percentages, and intergroup differences were evaluated by Chi-square test or Fisher’s exact test if more than 20% of cells had an expected count < 5. In cases where the overall Chi-square test was significant (*P* < 0.05), pairwise comparisons were performed using Fisher’s exact test with Bonferroni correction. Spearman’s correlation analysis was applied to examine the relationships between plasma RSPO2 concentrations and clinical parameters. Multivariate logistic regression analysis was used to assess the associations of RSPO2 with the likelihood of belonging to a higher kidney disease risk category. To control for potential confounding factors, which influence kidney disease progression and/or RSPO2 levels, including medications known to affect albuminuria and renal function, we constructed sequential models with incremental adjustments: Model 1 was unadjusted; Model 2 was adjusted for age and sex; Model 3 was further adjusted for BMI, drinking, history of hypertension, diabetes duration, DBP, and metformin use; Model 4 (the fully adjusted model) included all covariates from Model 3 plus the use of SGLT2 inhibitors, DPP-4 inhibitors, GLP-1R agonists, and RAS inhibitors. The results of these models are presented as odds ratios (ORs) with 95% confidence intervals (CIs).

## 3 Results

### 3.1 Clinical characteristics of the study participants

**Table 1** shows the clinical characteristics of participants in the four groups. Compared to the Low Risk and Moderately Increased Risk group, the Very High Risk group demonstrated significantly lower eGFR (29.25 ± 15.81 vs. 102.55 ± 12.67 and 97.97 ± 18.66 ml/min/1.73 m^2^, respectively; both *P* < 0.001) and significantly higher levels of UACR (1920.66 [570.82-3435.33] vs. 7.07 [4.82-17.21] and 76.79 [48.80-116.44] mg/g, respectively; both *P* < 0.001), Urea (11.89 [9.00-18.33] vs. 5.24 [4.30-6.30] and 5.54 [4.61-6.23] mmol/L, respectively; both *P* < 0.001) and Scr (186.85 [137.95-320.70] vs. 65.50 [54.95-75.95] and 65.20 [58.80-80.60] μmol/L, respectively; both *P* < 0.001).

**Table 1:** Clinical and laboratory characteristics of the study participants.

Characteristic	Low risk	Moderately increased risk	High risk	Very high risk
N	29	30	18	44
Age (years)	53.72 ± 13.79	52.47 ± 12.52	52.22 ± 13.97	56.41 ± 10.03
Male (%)	68.97% (20)	66.67% (20)	50.00% (9)	59.09% (26)
BMI (kg/m^2^)	24.74 ± 3.37	26.91 ± 3.20	25.56 ± 3.40	25.24 ± 2.81
Drinking (%)	41.38% (12)	43.33% (13)	27.78% (5)	22.73% (10)
Smoking (%)	27.59% (8)	43.33% (13)	33.33% (6)	31.82% (14)
History of hypertension (%)	48.28% (14)	60.00% (18)	61.11% (11)	93.18% (41)^bde^
Diabetes duration (years)	5.00 (3.00-12.00)	7.00 (2.50-10.50)	7.50 (2.75-13.25)	10.00 (6.00-15.00)
UACR (mg/g)	7.07 (4.82-17.21)	76.79 (48.80-116.44)^b^	752.02 (367.54 -1156.98)^bd^	1920.66 (570.82 -3435.33)^bd^
eGFR (ml/min/1.73m^2^)	102.55 ± 12.67	97.97 ± 18.66	86.17 ± 25.07	29.25 ± 15.81^bdf^
Urea (mmol/L)	5.24 (4.30-6.30)	5.54 (4.61-6.23)	5.99 (5.01-7.98)	11.89 (9.00-18.33)^bdf^
Scr (μmol/L)	65.50 (54.95-75.95)	65.20 (58.80-80.60)	83.40 (61.63-89.95)	186.85 (137.95-320.70)^bdf^
UA (μmol/L)	338.80 (280.15-403.90)	321.90 (254.05-402.30)	320.95 (275.60-376.45)	395.60 (350.75-460.93)^ace^
SBP (mm Hg)	128.24 ± 14.66	133.27 ± 15.41	128.89 ± 20.25	137.73 ± 18.55
DBP (mm Hg)	79.69 ± 10.43	85.27 ± 12.34	82.00 ± 15.17	79.11 ± 13.01
FBG (mmol/L)	6.97 (5.37-8.59)	6.65 (5.50-9.92)	6.87 (5.72-11.25)	6.23 (5.14-7.52)
HbA1c (%)	8.00 (6.55-9.35)	7.10 (6.15-10.43)	8.50 (6.48-10.65)	6.55 (6.00-7.65)^ae^
TG (mmol/L)	1.50 (0.98-3.17)	2.07 (1.37-3.62)	2.78 (1.32-6.40)	1.59 (1.21-2.48)
TC (mmol/L)	4.65 (3.71-5.56)	4.20 (3.51-5.51)	4.60 (3.88-5.67)	4.30 (3.43-6.07)
HDL-C (mmol/L)	1.03 ± 0.26	1.00 ± 0.28	1.09 ± 0.35	1.13 ± 0.30
LDL-C (mmol/L)	2.36 (1.32-3.16)	1.86 (1.50-2.59)	2.12 (1.59-2.75)	2.01 (1.35-2.99)
SGLT2 inhibitor usage (%)	37.93% (11)	50.00% (15)	72.22% (13)	43.18% (19)
Metformin usage (%)	44.83% (13)	50.00% (15)	61.11% (11)	18.18% (8)^ce^
α-Glucosidase inhibitor usage (%)	24.14% (7)	13.33% (4)	22.22% (4)	2.27% (1)^a^
Meglitinide usage (%)	3.45% (1)	10.00% (3)	11.11% (2)	18.18% (8)
DPP-4 inhibitor usage (%)	10.34% (3)	3.33% (1)	5.56% (1)	2.27% (1)
Sulfonylurea usage (%)	3.45% (1)	6.67% (2)	0.00% (0)	0.00% (0)
GLP-1 agonist usage (%)	10.34% (3)	26.67% (8)	5.56% (1)	2.27% (1)^c^
Insulin usage (%)	31.03% (9)	23.33% (7)	22.22% (4)	36.36% (16)
RAS inhibitor usage(%)	27.59% (8)	53.33% (16)	61.11% (11)	63.64% (28)^a^

The data are expressed as means ± SD or median with interquartile range or percentages. ^a^*P* < 0.01 compared with Low Risk; ^b^*P* < 0.001 compared with Low Risk; ^c^*P* < 0.01 compared with Moderately Increased Risk; ^d^*P* < 0.001 compared with Moderately Increased Risk; ^e^*P* < 0.01 compared with High Risk; ^f^*P* < 0.001 compared with High Risk. BMI, body mass index; UACR, urinary albumin/creatinine ratio; eGFR, estimated glomerular filtration rate; Scr, serum creatinine; UA, Uric acid; SBP, systolic blood pressure; DBP, diastolic blood pressure; FBG, fasting blood glucose; HbA1c, hemoglobin A1c; TC, total cholesterol; TG, triglyceride; HDL-C, high-density lipoprotein cholesterol; LDL-C, low-density lipoprotein cholesterol; SGLT2, sodium-glucose linked transporter 2; DPP-4, dipeptidyl peptidase-4; GLP-1, glucagon-like peptide-1; RAS, renin-angiotensin systeml.

UA levels were also significantly elevated in the Very High Risk group (395.60 [350.75-460.93] μmol/L) relative to the Low (338.80 [280.15-403.90] μmol/L, *P* < 0.01), Moderately Increased (321.90 [254.05-402.30] μmol/L, *P* < 0.01), and High Risk (320.95 [275.60-376.45] μmol/L, *P* < 0.01) groups. History of hypertension was markedly more prevalent in the Very High Risk group (93.18%) compared to all other groups (48.28%, *P* < 0.001, 60.00%, *P* < 0.001, and 61.11%, *P* < 0.01, respectively).

Regarding glycemic control and medication use, HbA1c was significantly lower in the Very High Risk group (6.55 [6.00-7.65]%) than in the Low (8.00 [6.55-9.35]%, *P* < 0.01) and High Risk (8.50 [6.48-10.65]%, *P* < 0.01) groups, while metformin usage was significantly lower in the Very High Risk group (18.18%) compared to the Moderately Increased (50.00%, *P* < 0.01) and High Risk (61.11%, *P* < 0.01) groups. The use of α-glucosidase inhibitors was significantly lower in the Very High Risk group than in the Low Risk group (2.27% vs. 24.14%, *P* < 0.01), and GLP-1 agonist usage was significantly lower in the Very High Risk group compared to the Moderately Increased Risk group (2.27% vs. 26.67%, *P* < 0.01).

### 3.2 Circulating RSPO2 levels increased across the kidney disease risk categories

**Figure 1:**
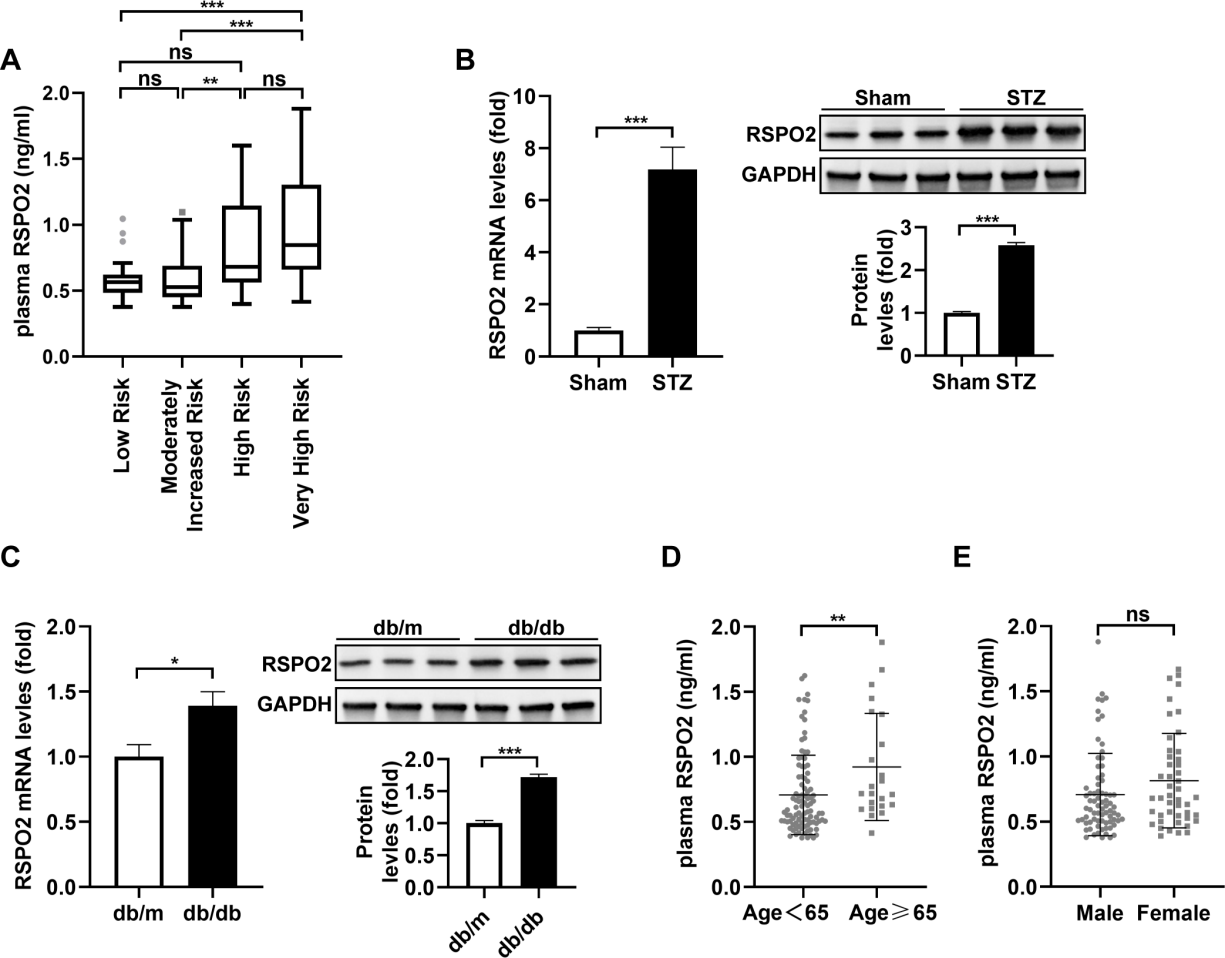
Plasma RSPO2 concentration in stratified subgroups from T2DM patients and RSPO2 expression in kidney tissue from DKD mouse models. **(A)** Plasma RSPO2 concentrations in indicated groups. mRNA and protein expressions of RSPO2 in kidney tissue from STZ-induced diabetic **(B)** and db/db mice **(C)**. Plasma RSPO2 concentrations in the study subjects, stratified by age **(D)** and sex **(E)**. For B and C, n = 6 mice per group. ***P < 0.001, **P < 0.01, *P < 0.05, ns, not significant.

Concentrations were significantly higher in the Very High Risk group (0.85[0.66-1.30]) compared to both the Low Risk (0.57[0.48-0.62] ng/mL, *P* < 0.001) and the Moderately Increased Risk (0.53[0.45-0.69] ng/mL, *P* < 0.001) groups. Additionally, RSPO2 levels in the High Risk group (0.68[0.56-1.15] ng/mL) were also elevated relative to the Low Risk group (, *P* < 0.01). No significant differences were observed between the Low Risk and Moderately Increased Risk groups (**Figure 1A**).

To explore whether the increased circulating RSPO2 levels in DKD are related to the renal RSPO2 expression, we established mouse models of T1DM and T2DM, respectively, and detected the expression of RSPO2 in kidney tissue. The results showed a significant elevation in RSPO2 levels in both STZ-induced diabetic (**Figure 1B**) and db/db (**Figure 1C**) mice. These findings suggest that kidney-derived RSPO2 may contribute to the pathological process of DKD.

Furthermore, when patients were stratified by age (< 65 vs. ≥ 65 years), plasma RSPO2 concentrations were significantly higher in the older group (0.92 ± 0.41 ng/mL) compared to the younger group (0.71 ± 0.30 ng/mL; *P* < 0.01) (**Figure 1D**), while no significant difference was identified between males and females (**Figure 1E**).

### 3.3 The relationships between plasma RSPO2 and other parameters.

**Figure 2:**
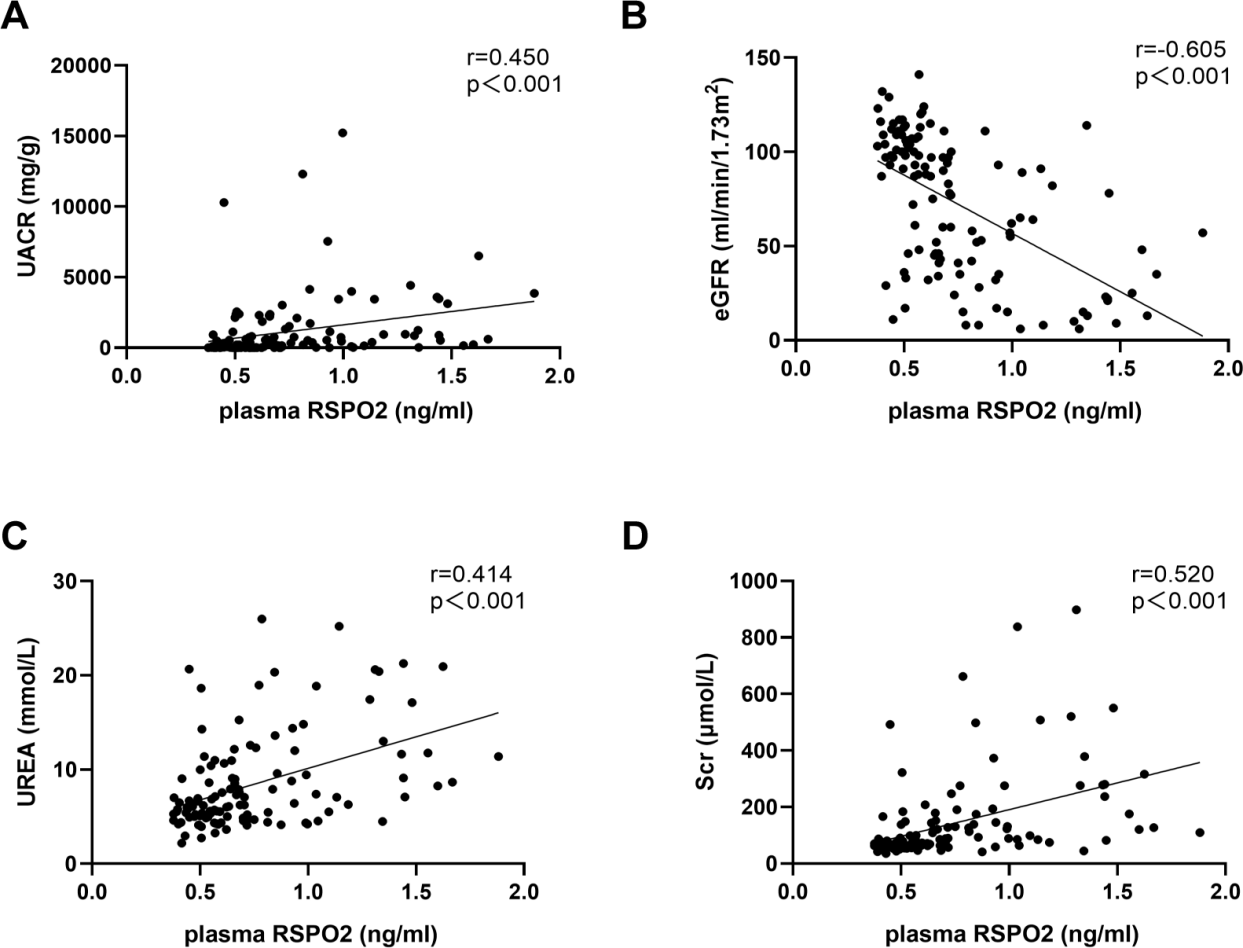
Scatter plots showing the correlation of plasma RSPO2 concentrations with UACR, eGFR, Urea, and Scr. **(A)** The correlation between plasma RSPO2 concentrations and UACR. **(B)** The correlation between plasma RSPO2 concentrations and eGFR. **(C)** The correlation between plasma RSPO2 concentrations and Urea. **(D)** The correlation between plasma RSPO2 concentrations and Scr. UACR, urinary albumin/creatinine ratio; eGFR, estimated glomerular filtration rate; Scr, serum creatinine.

To further elucidate the relationship between plasma RSPO2 concentration and renal function impairment in DKD, we performed Spearman’s correlation analysis. As shown in **Table 2**, plasma RSPO2 levels were positively correlated with age (*r* = 0.249, *P* = 0.006), history of hypertension (*r* = 0.399, *P* < 0.001), diabetes duration (*r* = 0.231, *P* = 0.011), UACR (*r* = 0.450, *P* < 0.001, **Figure 2A**), Urea (*r* = 0.414, *P* < 0.001, **Figure 2C**), Scr (*r* = 0.520, *P* < 0.001, **Figure 2D**), and HDL-C (*r* = 0.231, *P* = 0.011), and negatively correlated with eGFR (*r* = -0.605, *P* < 0.001, **Figure 2B**), drinking (*r* = -0.274, *P* = 0.002), smoking (*r* = -0.194, *P* = 0.033), DBP (*r* = -0.235, *P* = 0.010), and metformin usage (*r* = -0.309, *P* < 0.001). After adjusting for age, the correlations with UACR, Urea, Scr, eGFR, drinking, history of hypertension, DBP, and metformin usage remained statistically significant (all *P* < 0.05, **Table 2**). These results indicate that plasma RSPO2 is closely and consistently associated with impaired renal function and increased albuminuria in patients with type 2 diabetes.

**Table 2:** Spearman correlation coefficient of factors related to plasma RSPO2 concentrations.

Characteristic	Plasma RSPO2		Plasma RSPO2 (age-adjusted)	
	*r*	*P-*value	*r*	*P-*value
Age (years)	0.249	0.006	—	—
Male (%)	0.157	0.085	0.139	0.131
BMI (kg/m^2^)	-0.173	0.057	-0.147	0.110
Drinking (%)	-0.274	0.002	-0.217	0.017
Smoking (%)	-0.194	0.033	-0.133	0.146
History of hypertension (%)	0.399	<0.001	0.317	<0.001
Diabetes duration (years)	0.231	0.011	0.126	0.171
UACR (mg/g)	0.450	<0.001	0.288	0.001
eGFR (ml/min/1.73m^2^)	-0.605	<0.001	-0.527	<0.001
Urea (mmol/L)	0.414	<0.001	0.430	<0.001
Scr (μmol/L)	0.520	<0.001	0.440	<0.001
UA (μmol/L)	0.113	0.215	0.115	0.212
SBP (mm Hg)	0.098	0.283	0.076	0.409
DBP (mm Hg)	-0.235	0.010	-0.184	0.044
FBG (mmol/L)	-0.150	0.100	-0.164	0.073
HbA1c (%)	-0.115	0.208	-0.118	0.198
TG (mmol/L)	-0.155	0.090	-0.058	0.531
TC (mmol/L)	-0.027	0.772	0.009	0.921
HDL-C (mmol/L)	0.231	0.011	0.160	0.081
LDL-C (mmol/L)	0.031	0.738	0.057	0.538
SGLT2 inhibitor usage (%)	0.045	0.624	0.015	0.869
Metformin usage (%)	-0.309	<0.001	-0.199	0.029
α-Glucosidase inhibitor usage (%)	-0.085	0.353	-0.095	0.301
Meglitinide usage (%)	0.089	0.333	0.049	0.596
DPP-4 inhibitor usage (%)	0.038	0.678	0.045	0.624
Sulfonylurea usage (%)	-0.084	0.357	-0.082	0.371
GLP-1 agonist usage (%)	-0.177	0.052	-0.112	0.223
Insulin usage (%)	-0.005	0.955	-0.040	0.667
RAS inhibitor usage(%)	0.112	0.223	0.044	0.635

The bold values indicate the *P*-value which were < 0.05. BMI, body mass index; UACR, urinary albumin/creatinine ratio; eGFR, estimated glomerular filtration rate; Scr, serum creatinine; UA, Uric acid; SBP, systolic blood pressure; DBP, diastolic blood pressure; FBG, fasting blood glucose; HbA1c, hemoglobin A1c; TC, total cholesterol; TG, triglyceride; HDL-C, high-density lipoprotein cholesterol; LDL-C, low-density lipoprotein cholesterol; SGLT2, sodium-glucose linked transporter 2; DPP-4, dipeptidyl peptidase-4; GLP-1, glucagon-like peptide-1; RAS, renin-angiotensin system.

### 3.4 Correlation of plasma RSPO2 concentration with the risk of adverse DKD prognosis

To determine whether plasma RSPO2 concentration is independently associated with the risk of a worse kidney disease prognosis in T2DM, we conducted multivariate logistic regression analyses. Recent research has identified RSPO2 leads to adipose tissue hypertrophy and insulin resistance (13), therefore, we included BMI and diabetes duration in our models. Furthermore, alcohol consumption, history of hypertension, DBP, and metformin usage were listed as covariates because they were significantly correlated with RSPO2 in our cohort. Medication that affects renal function was also included, including SGLT2 inhibitor, DPP-4 inhibitor, GLP-1R agonists, and renin-angiotensin system (RAS) inhibitors. As shown in **Table 3**, an increase in RSPO2 remained independently associated with increased odds of belonging to the High Risk category (OR: 12.210, 95% CI: 2.231-66.829, *P* = 0.004) and the Very High Risk category (OR: 18.749, 95% CI: 4.236-82.983, *P* < 0.001) even after adjusting for age, sex, BMI, drinking, hypertension history, diabetes duration, DBP, and usage of medication. These results robustly demonstrate that elevated plasma RSPO2 concentration is an independent predictor of a more adverse kidney disease prognosis, as defined by the KDIGO risk stratification.

**Table 3:** Association of plasma RSPO2 concentrations with DKD by multivariate logistic regression analysis.

Model	High risk		Very high risk	
	OR (95% CI)	*P*-value	OR (95% CI)	*P*-value
Model 1	6.024 (1.632 - 22.233)	**0.007**	17.250 (5.297 - 56.180)	**<0.001**
Model 2	7.317 (1.780 - 30.076)	**0.006**	18.627 (5.381 - 64.482)	**<0.001**
Model 3	11.159 (2.222 - 56.048)	**0.003**	15.721 (3.729 - 66.269)	**<0.001**
Model 4	12.210 (2.231 - 66.829)	**0.004**	18.749 (4.236 - 82.983)	**<0.001**

Model 1, unadjusted.

Model 2, adjusted by age, sex.

Model 3, adjusted by age, sex, BMI, drinking, history of hypertension, diabetes duration, DBP, and metformin usage.

Model 4, adjusted by age, sex, BMI, drinking, history of hypertension, diabetes duration, DBP, metformin usage, SGLT2 inhibitor usage, DPP-4 inhibitor usage, GLP-1 agonist usage, and RAS inhibitor usage.

The bold values indicate the *P*-values which were < 0.05. BMI, body mass index; DBP, diastolic blood pressure; SGLT2, sodium-glucose linked transporter 2; DPP-4, dipeptidyl peptidase-4; GLP-1, glucagon-like peptide-1; RAS, renin-angiotensin system.

## 4 Discussion

This study is the first to systematically investigate the association between RSPO2 and DKD. We found that in patients with T2DM, plasma RSPO2 levels increased significantly with worsening prognosis risk strata, which integrate UACR and eGFR, correlated strongly with renal injury parameters, and emerged as an independent predictor of a more adverse kidney disease prognosis, as defined by the KDIGO risk stratification.

Plasma RSPO2 levels exhibited a gradational increase across the kidney disease prognosis risk spectrum. Notably, levels were already significantly elevated in the Very High Risk group compared to the Low Risk group. Importantly, according to KDIGO criteria, individuals in the Low Risk category (eGFR ≥60 mL/min/1.73 m² and UACR <30 mg/g) are not considered to have chronic kidney disease if no other markers of kidney damage are present. The fact that RSPO2 levels were already markedly higher in patients with overt DKD (Very High Risk) compared to this group without CKD, and showed a progressive rise with increasing kidney disease prognosis risk, suggestions that RSPO2 may participate in the development and progression of DKD. Next, we examined RSPO2 expression in established preclinical models of DKD. We employed both STZ-induced diabetic mice, a model of T1DM and early-stage DKD (23), and db/db mice, a genetic model of T2DM with progressive DKD features (24). In both models, RSPO2 mRNA and protein levels were elevated in kidney tissues. This indicates that abnormal RSPO2 expression occurs not only in the circulation of DKD patients but also locally in diabetic kidneys, potentially promoting DKD progression via paracrine and/or autocrine mechanisms.

Further age-stratified analysis showed that plasma RSPO2 concentrations were significantly higher in patients aged ≥ 65 years than in those aged < 65 years. This observation is consistent with our correlation analysis, which identified a significant positive correlation between plasma RSPO2 levels and age. Since aging is a recognized risk factor for DKD (25, 26), these findings suggest that plasma RSPO2 may be linked to the synergistic effects of age-related decline in renal function and diabetic kidney injury. A previous study reported that serum RSPO2 levels were significantly elevated in male individuals with insulin resistance compared to their insulin-sensitive counterparts, whereas no such difference was observed in females. Additionally, in males, serum RSPO2 levels were negatively correlated with the glucose infusion rate and positively correlated with both visceral fat area and maximal adipocyte size, whereas these associations were absent in female subjects (13). In light of these observations, we also incorporated a sex-stratified analysis. Our results revealed no significant difference in plasma RSPO2 concentrations between male and female diabetic patients, nor any significant association between plasma RSPO2 levels and sex.

Correlation analysis showed that plasma RSPO2 levels were positively correlated with UACR, Urea, and Scr, and negatively correlated with eGFR. UACR is an early indicator sensitive in the damage of glomerular filter barrier (27–29), while Scr, Urea, and eGFR are classical markers of renal functional impairment (20, 30). The close relationship of RSPO2 with these parameters suggests that RSPO2 may play an important role in the core pathology of DKD, like glomerular damage and poor kidney function. It is notable that plasma RSPO2 levels increased progressively across the KDIGO risk strata, a composite clinical prognostic tool that integrates both UACR and eGFR, together, these observations heighten the potential of RSPO2 as an early and integrative biomarker of identifying individuals at risk of progressive DKD.

Among the RSPO family, RSPO1 has been confirmed as circulating biomarker for obesity and insulin resistance (12), and obese patients carry the same mutations in RSPO1 (31). Silencing RSPO1 alleviates renal fibrosis in obese mice (17). Higher protein level in plasma of RSPO3 are associated with increased risk of T2DM (32). In mild obesity-related diabetes, RSPO3 is significantly associated with incident heart failure (33), and recombinant RSPO3 treatment ameliorated cardiac dysfunction in diabetic mice (34). RSPO3 regulates abnormal differentiation of small intestinal epithelial cells in diabetes (35), and has been identified to be associated with CKD in individuals with dysglycemia (18). Besides, hepatic stellate cells selective deletion of RSPO3 exacerbates alcohol-associated and metabolic dysfunction-associated steatotic liver disease (6). The role of RSPO4 in metabolism is initially seen in regulating bone deterioration caused by excessive phosphorus intake (36). The previous research on RSPO2 has primarily focused on tumors (37–40), limb development (5), and osteoarthritis (41, 42). In recent years, research suggests that RSPO2 plays an important role in fat metabolism, insulin resistance and atherosclerosis (13–16). Our multivariate logistic regression analysis revealed that a higher plasma RSPO2 concentration is independently associated with an increased likelihood of being classified into the High and Very High kidney disease prognosis risk categories. This significant associated persisted even after comprehensive adjustments for age, sex, BMI, alcohol consumption, history of hypertension, diabetes duration, DBP, metformin use, and medications that affect renal function. These findings suggest that elevated circulating RSPO2 levels may serve as a robust indicator of a worse kidney disease prognosis in T2DM. Our study is the first to unveil this clinical association between RSPO2 and DKD prognosis, thereby providing novel evidence supporting the role of the RSPO family in metabolism and its related renal complications.

Our research has several limitations. First, the cross-sectional design cannot establish a causal relationship between plasma RSPO2 levels and DKD. Prospective cohorts are needed to validate the predictive value of RSPO2. Second, this study was conducted at a single center with a relatively homogeneous population, which may limit the generalizability of our findings to other ethnic or geographic groups. Future multi-center studies involving more diverse cohorts are warranted to validate and extend our conclusions. Third, there may have been some selection bias because of the relatively small sample.

In summary, this study demonstrates that in patients with T2DM, plasma RSPO2 levels are significantly elevated in those with a worse kidney disease prognosis, strongly correlate with renal injury, and serve as an independent predictor for more severe KDIGO prognostic categories. These findings broaden the scope of RSPO family research in metabolic diseases and provide new molecular insights for early identification of high-risk individuals and targeted therapy of DKD.

## Data Availability

The original contributions presented in the study are included in the article/supplementary material. Further inquiries can be directed to the corresponding authors.
